# Supplementary material for: Validation of the Arabic Maternal postpartum quality of life questionnaire among Lebanese women: A cohort study
Source: PLoS One. 2023 Sep 28;18(9):e0291826. doi: 10.1371/journal.pone.0291826 (PMC10538779; doi:10.1371/journal.pone.0291826)
Supplement: S1 File — (PDF) [file pone.0291826.s001.pdf]

إستبيان جودة الحياة بعد الولادة للأمهات (الشهر الأول بعد الولادة)

الجزء الأول: لكل من الأسئلة اللاحقة، يرجى إختيار الجواب الذي يصف جيدا" مدى رضاك عن هذا الجزء من حياتك.

الرجاء الإجابة بوضع دائرة حول رقم الإجابة. لا يوجد جواب صحيح أو خطأ.

1- غير راضية بشدة

2- غير راضية نوعا" ما

3- غير راضية قليلا"

4- راضية قليلا"

5- راضية نوعا" ما

6- راضية بشدة

<u>ما مدى رضاك عن؟</u>

1. صحتك	1	2	3	4	5	6
2. مقدار الألم الذي تعانينه	1	2	3	4	5	6
3. مقدار الطاقة اليومية لديك للقيام بنشاطك اليومي	1	2	3	4	5	6
4. مقدار التحكم في حياتك اليومية	1	2	3	4	5	6
5. قدرتك على الاعتناء بنفسك دون مساعدة	1	2	3	4	5	6
6. مظهرك الخارجي	1	2	3	4	5	6
7. ثدياك	1	2	3	4	5	6
8. جرح الولادة	1	2	3	4	5	6
9. حياتك الجنسية	1	2	3	4	5	6
10. راحة بالك	1	2	3	4	5	6
11. سعادتك بشكل عام	1	2	3	4	5	6
12. حياتك بشكل عام	1	2	3	4	5	6
13. مقدار الهموم في حياتك	1	2	3	4	5	6
14. الدعم العاطفي الذي تتلقينه من:						
a. من زوجك/ شريكك	1	2	3	4	5	6
b. عائلتك	1	2	3	4	5	6

c. أصدقاؤك أو الناس الآخرين	1	2	3	4	5	6
15. علاقتك بزوجك أو شريكك	1	2	3	4	5	6
16. قدرتك على تلبية مسؤولياتك العائلية	1	2	3	4	5	6
17. صحة طفلك	1	2	3	4	5	6
18. المساعدة في العناية بطفلك وأولادك الآخرين	1	2	3	4	5	6
19. الوقت الهخصص للأولاد	1	2	3	4	5	6
20. الوقت المخصص للأعمال المنزلية	1	2	3	4	5	6
21. الوقت المخصص للأصدقاء/ الأقارب	1	2	3	4	5	6
22. الوقت المخصص للزوج /الشريك	1	2	3	4	5	6
23. الوقت المخصص لنفسك	1	2	3	4	5	6
24. القدرة على إطعام طفلك الجديد	1	2	3	4	5	6
25. صحة زوجك / شريكك	1	2	3	4	5	6
26. روتين حياتك اليومية	1	2	3	4	5	6
27. منزلك/شقتك/مكانك الذي تعيشين فيه	1	2	3	4	5	6
28. الحي الذي ثعيشين فيه	1	2	3	4	5	6
29. استقلالك المالي	1	2	3	4	5	6
30. قدرتك على تلبية المتوجبات المالية	1	2	3	4	5	6
31. توفر الرعاية الطبية لك	1	2	3	4	5	6
32. توفر وسيلة تنقل لك	1	2	3	4	5	6
33. أوضاعك المعيشية في المنزل						
a. ممتلكاتك المادية	1	2	3	4	5	6
b. قدرتك الاقتصادية أو المالية	1	2	3	4	5	6
c. بيئتك/محيطك بشكل عام (لا صريخ، منازعات، شجارات)	1	2	3	4	5	6
34. الوظيفة/العمل						
a. وظيفة زوجك	1	2	3	4	5	6
b. وظيفتك	1	2	3	4	5	6

الملحق "ب"

الجزء الثاني: لكل من الأسئلة اللاحقة، يرجى إختيار الجواب الذي يصف جيدا" مدى أهمية هذا الجزء من حياتك.

الرجاء الإجابة بوضع دائرة حول رقم الإجابة. لا يوجد جواب صحيح أو خطأ.

1. غير مهم بشدة
2. غير مهم نوعا" ما
3. غير مهم قليلا"
4. مهم قليلا"
5. مهم نوعا" ما
6. مهم بشدة

<u>ما مدى أهمية ما يلي بالنسبة لك؟</u>

1. صحتك	1	2	3	4	5	6
2.مقدار الألم الذي تعانينه	1	2	3	4	5	6
3.مقدار الطاقة اليومية لديك للقيام بنشاطك اليومي	1	2	3	4	5	6
4.مقدار التحكم في حياتك اليومية	1	2	3	4	5	6
5.قدرتك على الاعتناء بنفسك دون مساعدة	1	2	3	4	5	6
6.مظهرك الخارجي	1	2	3	4	5	6
7.ثدياك	1	2	3	4	5	6
8.جرح الولادة	1	2	3	4	5	6
9.حياتك الجنسية	1	2	3	4	5	6
10.راحة بالك	1	2	3	4	5	6
11.سعادتك بشكل عام	1	2	3	4	5	6

12.حياتك بشكل عام	1	2	3	4	5	6
13.مقدار الهموم في حياتك	1	2	3	4	5	6
14.الدعم العاطفي الذي تتلقينه من:						
c. من زوجك/ شريكك	1	2	3	4	5	6
d. عائلتك	1	2	3	4	5	6
e. أصدقاؤك أو الناس الآخرين	1	2	3	4	5	6
15. علاقتك بزوجك أو شريكك	1	2	3	4	5	6
16.قدرتك على تلبية مسؤولياتك العائلية	1	2	3	4	5	6
17.صحة طفلك	1	2	3	4	5	6
18.المساعدة في العناية بطفلك وأولادك الآخرين	1	2	3	4	5	6
19.الوقت الهخصص للأولاد	1	2	3	4	5	6
20.الوقت المخصص للأعمال المنزلية	1	2	3	4	5	6
21.الوقت المخصص للأصدقاء/ الأقارب	1	2	3	4	5	6
22.الوقت المخصص للزوج /الشريك	1	2	3	4	5	6
23.الوقت المخصص لنفسك	1	2	3	4	5	6
24.القدرة على إطعام طفلك الجديد	1	2	3	4	5	6
25.صحة زوجك / شريكك	1	2	3	4	5	6
26.روتين حياتك اليومية	1	2	3	4	5	6
27.منزلك/شقتك/مكانك الذي تعيشين فيه	1	2	3	4	5	6
28.الحي الذي ثعيشين فيه	1	2	3	4	5	6

29.استقلالك المالي	1	2	3	4	5	6
30.قدرتك على تلبية المتوجبات المالية	1	2	3	4	5	6
31.توفر الرعاية الطبية لك	1	2	3	4	5	6
32.توفر وسيلة تنقل لك	1	2	3	4	5	6

33.أوضاعك المعيشية في المنزل

f. ممتلكاتك المادية	1	2	3	4	5	6
g. قدرتك الاقتصادية أو المالية	1	2	3	4	5	6
h. بيئتك/محيطك بشكل عام (لا صريخ، منازعات، شجارات)	1	2	3	4	5	6

34. الوظيفة/العمل

i. وظيفة زوجك	1	2	3	4	5	6
j. وظيفتك	1	2	3	4	5	6
