# Supplementary material for: Validation of the Arabic Maternal postpartum quality of life questionnaire among Lebanese women: A cohort study
Source: PLoS One. 2023 Sep 28;18(9):e0291826. doi: 10.1371/journal.pone.0291826 (PMC10538779; doi:10.1371/journal.pone.0291826)
Supplement: Attachment — Submitted filename: Response to Reviewers.docx [file pone.0291826.s003.docx]

**DEPARTMENT OF PEDIATRICS**

**AND ADOLESCENT MEDICINE**

Date: July 3, 2023

Dear Editor,

We would like to submit our revised manuscript [PONE-D-23-06864] entitled “Validation of the Arabic Maternal Postpartum Quality of Life Questionnaire Among Lebanese women: A Cohort Study” for publication in *PLOS ONE*. All changes have been highlighted in yellow in the revised tracked copy. We are very thankful for the constructive comments of the reviewers which we hope will improve our manuscript.

Below, please find our response to each comment.

**Journal Requirements:**

1. **Please ensure that your manuscript meets PLOS ONE’s style requirements, including those for file naming.**

Answer: Done. The manuscript was revised in accordance with PLOS ONE’s style requirements, including file naming.

2. **Minimal data set: Please upload your study’s minimal data set as either Supporting Information files or to a stable public repository.**

Answer: We uploaded our minimal deidentified data set as Supporting Information file (S2 File).

3. **Data Availability: Please describe the changes you make to Data Availability Statement in your cover letter.**

Answer: We submitted our anonymized dataset to PLOS ONE with the revised manuscript as Supporting Information File (S2 File). **Kindly update our Data Availability statement on our behalf to reflect this information**.

4. **Your Ethics statement should only appear in the Methods section of your manuscript. Please ensure that your ethics statement is included in your manuscript.**

Answer: We included the following ethics statement in the Methods section:

“*This study was approved by the Institutional Review Board of the American University of Beirut, reference number: PED.MN.16/SBS-2017-0450. Written informed consent was obtained from all participants”*

**Editor’s comments:**

- **Please remove the abbreviation from the title.**

Answer: We removed the abbreviation from the title.

**Response to Reviewer 1:**

Thank you for your constructive comments and the valuable review that helped us improve our manuscript. Please find below our reply to each comment.

1. **The sample size calculation needs to be specified.**

Answer: We added the following statement and references to the Methods/Statistical analysis section:

*“The sample size calculation for this study was based on the recommended ten participants for every question in instrument validation studies [14, 15]. Since the MAPP-QOL is composed of 39 items, we aimed to recruit at least 390 women. We inflated the sample size to 485 participants to account for a potential 25% loss to follow up.”*

2. **The statistical analysis section is limited to exploratory analysis only. The domains need to be rechecked using confirmatory factor analysis. Please add.**

Answer: Thank you for your comment. The reason we conducted Exploratory Factor Analysis is because the original English MAPP-QOL was validated in only 2 contexts (Brazilian and Persian) that are distinctly different from the Lebanese context. Hence, we wanted to explore how it would perform in our culture. Confirmatory Factor Analysis would be more suitable when the instrument has been well established to have good reliability and validity in most settings (e.g. The Beck Depression Inventory used to screen for depression and its severity in people over 13 years of age).

3. **A study flow needs to be added.**

Answer: We added a flow diagram (Figure 1).

4. **ABSTRACT/Results: Where are the validity test results reflected? Please add.**

Answer: Kindly refer to the below paragraph in the revised Abstract/Results:

*“The overall Arabic MAPP-QOL score was positively but weakly correlated with the Arabic Maternal Breastfeeding Evaluation Scale score (r = 0.177, p < 0.001), its Maternal Enjoyment/Role Attainment subscale score (r = 0.108, p = 0.023), and Lifestyle/Body Image subscale score (r = 0.286, p < 0.001). There was no significant association between the type of infant feeding at one month and the Arabic MAPP-QOL score (p = 0.932). Similarly, the Arabic MAPP-QOL score was not correlated with the participant’s age (r = 0.043, p = 0.362) or education (p = 0.451)”*

5. **ABSTRACT/Methods: What method was used to select the sample? What methods and software were used for statistical analysis?**

Answer: We revised the Abstract/Methods to describe how the sample was selected, and summarized the methods used in statistical analysis and the software as follows:

*“This instrument validation cohort study tested an adapted Arabic version of the MAPP-QOL questionnaire on a convenience sample of 485 healthy Lebanese postpartum women. The MAPP-QOL reliability and validity were investigated by conducting Exploratory Factor Analysis using Principal Component Analysis, and by correlating the participants’ MAPP-QOL scores with their scores on the Arabic Maternal Breastfeeding Evaluation Scale (MBFES-A), age, and education. All statistical analyses were done using SPSS version 23”*

6. **BACKGROUND: Line 88-90, page 5: Just because Lebanon does not have an Arab version, so do it? Is this the so-called research meaning? Please add clarification.**

Answer: Thank you for your comment. We added the following paragraph to the Background of the revised manuscript clarifying the existing knowledge gap and the need for an Arabic version of the MAPP-QOL:

*“There are few studies from the Arab world that assessed the QOL of Arab women during the postpartum period and its association with different health-related issues or conditions. None of these used the MAPP-QOL that is specific for this period [9-10]. The QOL instruments used in these studies included the Health-Related Quality of Life Scale [9], the single-item global QOL question [10], and the Chinese QOL instrument by Zhou, et al. the reliability and validity of which were not tested [8, 11]. Hence, there is a need for a valid instrument that can reliably assess the postpartum QOL in Arab women. In this study, we explored whether an Arabic version of the MAPP-QOL is a valid and reliable instrument to measure the different aspects of postpartum QOL in a cohort of healthy Lebanese women*.*”*

7. **BACKGROUND/Overall: 1) What was the basis of your topic? Could some research background be added on the quality of life of postpartum women in Lebanon or Arabia? 2) What is your research hypothesis?**

Answer: We addressed your comment about the basis of our topic in the following paragraph (similar to comment 6 above):

*“There are few studies from the Arab world that assessed the QOL of Arab women during the postpartum period and its association with different health-related issues or conditions. None of these used the MAPP-QOL that is specific for this period [9-10]. The QOL instruments used in these studies included the Health-Related Quality of Life Scale [9], the single-item global QOL question [10], and the Chinese QOL instrument by by Zhou, et al. the reliability and validity of which were not tested [8, 11]. Hence, there is a need for a valid instrument that can reliably assess the postpartum QOL in Arab women.”*

Our research hypothesis is summarized in the below statement:

*In this study, we explored whether an Arabic version of the MAPP-QOL is a valid and reliable instrument to measure the different aspects of postpartum QOL in a cohort of healthy Lebanese women*.*”*

8. **METHODS/Design and setting: Line 97-100, page 5: The description of the inclusion criteria should be more complete and clarifications such as “firstly” could be considered. Similarly, the exclusion criteria should be clearly written.**

Answer: We revised the statement on inclusion and exclusion criteria as suggested:

*“The inclusion criteria were: 1) delivery of a healthy term singleton newborn, 2) intention to breastfeed after delivery, 3) ability to read and write in Arabic. Exclusion criteria were: 1) women who had twins, 2) delivery before 37 weeks of gestation, 3) women diagnosed with a chronic disease, 4) not intending to breastfeed, 5) infant admission to the neonatal intensive care unit”*

9. **METHODS/Design and setting: Line 101-102, page 5: 1) Is it appropriate to select the sample only from a tertiary academic center in the capital city Beirut? Can the results of the study be extrapolated? I think it is under-represented. 2) Please add the relevant content of the ethical review.**

Answer: We agree with the kind reviewer that our sample is not representative of all Lebanese women. However, with the resources available to us, we could only recruit from our center which is the largest referrral academic center in Lebanon. We have addressed this limitation in the Discussion section as follows:

“*Our study’s main limitation is that most of the participants were recruited from a major tertiary care center which serves highly educated, middle- or high-income women from the capital city. Therefore, the findings of this study may not be generalizable to other Lebanese women with different socioeconomic or education status, or to other Arab women*”

We also added the following statement on the ethical review:

“*This study was approved by the Institutional Review Board of the American University of Beirut, reference number: PED.MN.16/SBS-2017-0450. Written informed consent was obtained from all participants”*

10. **METHODS/Measurement: Line 101-111, page 5-6: Most of this is duplicated with the background part. Please modify it.**

Answer: Thank you. We removed the redundant statements from the Measurement section of the revised manuscript as suggested.

11. **METHODS/Data collection: Line 145, page 7: “Arabic Maternal Breastfeeding Scale (MBFES-A)” How reliable or valid is the scale? Please provide a brief description of the entry and score for the MBFES-A.**

Answer: We added the follwoing statements that describe the reliability and validity of the MFFES-A, and its scoring:

“*The participants were also administered the Arabic Maternal Breastfeeding Evaluation Scale (MBFES-A) at one month. This 26-item instrument has been shown to be a reliable and valid tool to measure maternal perceived overall quality of the breastfeeding experience in Lebanese women, with a Cronbach’s alpha reliability coefficient of 0.87 [13]. The item scoring uses a 5-point Likert-type scale that ranges from 1 (strong disagreement) to 5 (strong agreement) resulting in a minimum score of 26 and a maximum of 130 points, with higher scores indicating more satisfaction with the breastfeeding experience.”*

12. **METHODS/Statistical analysis: Line 160, page 8: You only used exploratory factor analysis, what about confirmatory factor analysis?**

Answer: We addressed this comment in our reply to comment 2 above.

13. **METHODS/Statistical analysis: Line 168, page 8: The research hypothesis should be placed in the last paragraph of the introduction. The statistical analysis section states only the statistical analysis method.**

Answer: We agree with the kind reviewer about this point. Kindly note that the aim of this study is to explore whether the MAPP-QOL is reliable and valid in our context. This is now clearly stated in the last paragraph of the Background section.

*“In this study, we explored whether an Arabic version of the MAPP-QOL is a valid and reliable instrument to measure the different aspects of postpartum QOL in a cohort of healthy Lebanese women*.*”*

We revised the Statistical section so the reader is not confused about the research hypothesis regarding the use of the variables MBFES-A scores, participant age and education as follows:

*“The* *predictive validity of MAPP-QOL was tested in bivariate analyses by correlating the participants’ scores on MAPP-QOL with their MFBES-A scores, or with age using Pearson’s correlation coefficient r, and with education using t test. These three variables were chosen for this analysis because women who are more satisfied with their breastfeeding experience, are older in age, or are more educated may be more able to cope with the challenges of the postpartum period, and hence may have a better quality of life”*

14. **RESULTS: Line 117, page 8, Since 39 of the 485 people recruited dropped out, shouldn’t your final sample size be 446? What exactly is the value of “N” in the header of the Table? Please confirm.**

Answer: I believe the kind reviewer is referring to Table 1 which summarizes the baseline characteristics of the 485 recruited participants. In fact, the withdrawals were 37 and not 39 women. We corrected this error in the revised manuscript.

The reason we wrote N=485 is because we have data on their baseline charcterisitcs at enrolment, and the 37 withdrawals happened later during the study. It is usually expected of authors to report all available data at each time point of the study.

“N” refers to the total sample size, whereas ‘n’ refers to a subgroup of the total sample.

15. **RESULTS: Table 1, page 9: Please make sure that the sum of the components in the table is 100%.**

Answer: Thank you for alerting us to this point. We checked all values and added the missing values so that the sum of the components is 100%.

16. **RESULTS: Line 203, page 12: The Cronbach coefficient for the health dimension is less than 0.7, how do you solve it?**

Answer: Despite the low Cronbach coefficient of the Health dimension, we decided to keep this dimension for several reasons: 1) The health dimension and the items included in it are all health aspects that are important for postpartum women (e.g. wound, episiotomy, pain, etc..); 2) All items in this dimension have communaltites ranging between 0.338 and 0.580; 3) When we tried different scenarios in which items were deleted and the Factor loading was repeated, we got lower Cronbach alpha values. We even tried the scenario of 4 components instead of 5 and also got lower reliability. Hence we decided on keeping this dimension despite its low Cronbach coefficient because of the importance of its items to QOL in the postpartum period.

17. **RESULTS/Overall: 1) If there are no special requirements, it is recommended to use a tri-line table for all forms to make it more aesthetically pleasing. 2) The results section can list sub-headings for each section separately to make the logic clearer.**

Answer: Thank you. We formatted the Tables as suggested and in alignment with the journal’s requirements.

The Results section now has 3 subheadings to make it clearer: baseline charcterisitcs, reliability, external validity.

18. **DISCUSSION/ Overall: 1) The discussion section has too few references, please add relevant arguments. 2) The discussion section did not explain the interpretation of the results and subsequent processing methods. Please fully explain and compare the results section.**

Answer: Thank you for your valuable suggestions. We revised the Discussion section extensively, added more references to support relevant arguments. We also added more interpretations of the results and compared/contrasted findings with the literature. Kindly refer to the yellow-highlited sentences in the revised Discussion section.

19. **CONCLUSIONS: Line 265-266, page 14: So what is the relationship between the quality of life of women in the early postpartum period?**

Answer: Kindly note that the aim of our study was not to establish a relationship between QOL of postpartum women and breastfeeding or other varialbles. The aim was to explore its validity and reliability in our context. Satisfaction with breastfeeding as measured by the MBFES scores was used to establish the external validity of the MAPP-QOL. We have revised the conclusions for clarity as follows:

“*The Arabic MAPP-QOL has good psychometric properties. This instrument may be a useful tool for clinicians as well as researchers interested in measuring maternal postpartum QOL in the Arab contest. Further replication of our findings in other Arab contexts is needed*”

20. **The starting point and research theme of this article are innovative. However, the logic and English formulation need to be strengthened.**

Answer: Thank you. We revised the manuscript in light of all of your suggestions and comments, including the English language. We hope it meets your satisfaction now.

21. **Why is confirmatory factor analysis not directly used for QOLs with relatively stable structures?**

Answer: We addressed this comment above. Kindly refer to our reply to comment 2 and comment 12.

22. **The discussion section did not explain the results reasonably and was confusing. It is recommended to read more references to provide theoretical support.**

Answer: We revised the Discussion and provided more explanations of our findings with comparisons to the literature. Kindly refer to the yellow-highlighted sentences in the Discussion section.

23. **The typography of the text of the whole article is particularly messy, please pay attention to the alignment.**

Answer: Thank you for the alert. Done.

**Response to Reviewer 3:**

Thank you for the valuable time you put to review our manuscript. Please find below our reply to each comment.

1. **This is a very well-written and valuable paper. Some minor suggestions.**

Answer: Thank you very much. We greatly appreciate your feedback.

2. **The method of sampling should be added in method portion of manuscript.**

Answer: We added the following statements about the sampling method:

Abstract/Methods: “*This instrument validation cohort study tested an adapted Arabic version of the MAPP-QOL questionnaire on a* ***convenience sample*** *of 485 healthy Lebanese postpartum women*”

Methods/Design and setting: “*Participants were* ***consecutively recruited*** *from the postpartum ward of a tertiary care academic center in the capital city Beirut*”

3. **The communalities of all iems need to be provided alongside the factor loads.**

Answer: We added the communalities of all items to Table 2.

4. **Please include the Arabic version of the MAPP-QOL as a supplementary material for the accessible use of other researchers and clinicians.**

Answer: The Arabic MAPP-QOL is included as Supplementary file (S1 File).

5. **Why confirmatory factor analysis (CFA) has not been used?**

Answer: Thank you for your comment. The reason we conducted Exploratory Factor Analysis is because the original English MAPP-QOL was validated in only 2 contexts (Brazilian and Persian) that are distinctly different from the Lebanese context. Hence, we wanted to explore how would it perform in our culture. Confirmatory Factor Analysis would be more suitable when the instrument has been well established to have good reliability and validity in most settings (e.g. The Beck Depression Inventory used to screen for depression and its severity in people over 13 years of age).

6. **Have the face validity and content validity been performed after the forward-backward translation?**

Answer: Face and content validities were assessed by both authors before piloting. Both authors were in full agreement that the Arabic version had good face validity and content validity. The items in the questionnaire captured almost all facets of quality of life specific for the postpartum period such as pain, breasts, support, health issues, time for children, family, friends, employment, etc...Moreover, results of piloting the questionnaire on 20 women supported its face and content validities.

7. **Although the slope on the scree plot was leveling after fourth factor, it seems that the decision to retain five factors was to some extent arbitrary. Additional argument for such a decision could be obtained by employing some further procedures aimed at determining the optimal number of factors, such as Hornn’s parallel analysis or Velicer’s minimum average partial.**

Answer: We greatly appreciate your suggestion. The reason we chose five factors instead of 5 are based on the following reasons: 1) The Scree plot between the fourth and the fifth factor is going downward steeply whereas it is leveling after the fifth factor; 2) the total variance explained by 5 factors was 43.7% vs. 39.9% total variance explained by 4 factors.

As for your suggestion to conduct Hornn’s parallel analysis or Velicer’s minimum average partial, kindly note that we are not familiar with those tests. Hence we consulted with our biostatistician and another epidemiologist who is an expert in Instrument validation. Both consultants stated that it is not a must to do these tests, and the biostatistician stated that he had not run such tests before. Any further advice on this issue from the kind reviewer is very much appreciated.

We hope we have addressed all the comments of the kind reviewers in a clear manner, and revised the manuscript accordingly.

Thank you again for the thorough review of our paper.

Best wishes.

Sincerely,

Mona Nabulsi, MD, MSc

Professor of Clinical Pediatrics

Department of Pediatrics and Adolescent Medicine

Faculty of Medicine

American University of Beirut

Beirut-Lebanon

P.O.Box: 113-6044/C8

E-mail: [mn04@aub.edu.lb](mailto:mn04@aub.edu.lb)
