# Supplementary material for: Validation of the Arabic Maternal postpartum quality of life questionnaire among Lebanese women: A cohort study
Source: PLoS One. 2023 Sep 28;18(9):e0291826. doi: 10.1371/journal.pone.0291826 (PMC10538779; doi:10.1371/journal.pone.0291826)
Supplement: Attachment — Submitted filename: Response to Reviewers.docx [file pone.0291826.s004.docx]

**DEPARTMENT OF PEDIATRICS**

**AND ADOLESCENT MEDICINE**

Date: September 4, 2023

Dear Editor,

We would like to submit our revised manuscript [PONE-D-23-06864] entitled “Validation of the Arabic Maternal Postpartum Quality of Life Questionnaire Among Lebanese women: A Cohort Study” for publication in *PLOS ONE*. All changes have been highlighted in yellow in the revised tracked copy. We are very thankful for the editor and the reviewers for the time they dedicated to review our manuscript and provide us with their valuable comments.

Below, please find our response to each comment.

**Journal Requirements:**

1. **Please review your reference list to ensure that it is complete and correct. If you have cited papers that have been retracted, please include the rationale for doing so in the manuscript text, or remove these references and replace them with relevant current references. Any changes to the reference list should be mentioned in the rebuttal letter that accompanies your revised manuscript. If you need to cite a retracted article, indicate the article’s retracted status in the References list and also include a citation and full reference for the retraction notice.**

Answer: We reviewed our reference list meticulously and did not identify any article that was retracted. We would greatly appreciate specifying which article in our reference list the kind editor is referring to.

**Additional Editor’s comments:**

1. **Authorship update: Kindly note that we added the PhD candidate Ms. Nour Abou Khalil as a third author to the list of authors in the revised manuscript. Ms Abou Khalil conducted the Confirmatory Factor Analysis that was requested by Reviewer 2.**
2. **Abstract: Repeated sentence in Results section, please remove it. “All statistical analyses were done using SPSS version 23”.**

Answer: Done. Thank you for alerting us to this error.

3. **Results: In the validity section, Table 2, please add the total variation of the validated scale and the eigen values for all dimensions.**

Answer: We added the total variation of the scale and Eigen values of each component. They are summarized in a new table labelled as Table 2. The old Table 2 is now labelled as Table 3. We prefer not to merge the 2 tables but keep them separate for clarity.

**Table 2. Eigen values for the 5 components of the scale and the total variance.**

| **Component** | **Initial Eigen Values** | | |
| --- | --- | --- | --- |
|  | **Total** | **% of Variance** | **Cumulative %** |
| 1 | 8.570 | 21.975 | 21.975 |
| 2 | 2.878 | 7.380 | 29.355 |
| 3 | 1.998 | 5.123 | 34.477 |
| 4 | 1.807 | 4.633 | 39.111 |
| 5 | 1.374 | 3.523 | 42.633 |

4. **Validity: The results of the KMO and Bartlett test of sphericity should be presented in the validity section rather than the reliability section.**

Answer: We moved the sentence about the results of the KMO and Bartlett test of sphericity from the reliability section to the validity section next to the component analysis.

5. **Discussion: Additional references are needed in the Discussion to compare the findings with previous studies. This allows for a broader understanding of the results and their implications.**

Answer: In the 2nd paragraph of the Discussion, we comapred and contrasted the results of our instrument (the Arabic MAPP-QOL) to the oringinal English MAPP-QOL with respect to the subsacles and relocation of items explaining the relocations in relation to our context. We also compared our finding of the association between better quality of life and the participants’ satisfaction with their overall breastfeeding experience, maternal enjoyment and role attainment, and lifestyle and body image to the literature (References 13, 17, 18, 19). Moreover, we discussed the differences between our instrument and the English, Persian, and Brazilian MAPP-QOL in terms of sample size and breastfeeding (in the Discussion). Kindly note that in the Introduction, we discussed in detail the psychometrics of the English, Brazilian, and Persian versions of the MAPP-QOL. We also discussed in the Introduction the existing literature on the QOL of postpartum women in the Arabic context. We feel that re-discussing the comparisons made in the Introduction again in the Discussion would be redundant. To our knowledge, these MAPP-QOL versions are the only ones in the literature.

**Response to Reviewer #2:**

Thank you for the valuable time you put to review our manuscript. Please find below our reply to each comment.

1. **Abstract: Results: 1) Please delete the sentence “All statistical analyses were done using SPSS version 23”. 2) Please make the text more concise.**

Answer: We deleted the repeated sentence. We removed one long sentence from the Results to make the text more concise. After careful review of the remaining sentences, we felt that none of them could be deleted since they are summarizing the reliability and validity assessment of the scale.

2. **Background: Overall: Why was this study conducted in the Arab region? Could some research background be added on the quality of life of postpartum women in Lebanon or Aarbia?**

Answer: We added the below summary of the few available studies about QOL in postpartum Arab women to the Introduction:

“*There have been no studies from Lebanon that described the quality of life in postpartum women. Few studies from the Arab world assessed the QOL of postpartum Arab women and its association with different health-related issues or conditions [9-11]. A study from Saudi Arabia reported that 59.68% of postpartum women had probable postpartum depression, with an inverse correlation between postpartum depression and health-related quality of life scores measured by the Health-Related Quality of Life Scale [9]. In another cross-sectional study, Badr et al. recruited a convenience sample of Arabic-speaking postpartum women through Arab groups on Facebook and WhatsApp. They examined the associations between postpartum fatigue, depressive cognitions, life satisfaction, resourcefulness, and quality of life using the single-item global QOL question [10]. This study found significant correlations between postpartum fatigue and each of the three remaining variables. A third study from Egypt [11] assessed the quality of life in postpartum women in relation to the various modes of delivery using the Chinese QOL by Zhou, et al., the reliability and validity of which were not tested [8]. There were no significant differences in the QOL aspects between mothers who delivered vaginally and those who delivered via Cesarean section. To note, none of the previous studies used the MAPP-QOL that is specific for the postpartum period. In view of the importance of the postpartum period in a woman’s life, and its associated physical, emotional, and social changes, there is a need for a valid instrument that can reliably assess the postpartum QOL in Arab women*”

3. **Methods: Statistical Analysis, Pages 8-9, Line 172-179: After reading your reply, can you conduct confirmatory factor analysis on top of exploratory factor analysis? After all, exploratory factor analysis alone is not sufficient in the study of measurement characteristics of scales.**

Answer: We appreciate the reviewer’s thoughtful question regarding our choice to conduct Exploratory Factor Analysis (EFA) rather than Confirmatory Factor Analysis (CFA) on the Arabic-PPQOL scale. Our decision to initially conduct EFA was driven by the unique context and translation of the scale. Given the cultural and contextual variations, we aimed to explore which factor structure would align optimally with our data. EFA allowed us to identify underlying patterns and relationships among items without imposing any preconceived factor structure. Subsequently, we performed CFA to assess the adequacy of the hypothesized factor structure proposed by Hill et al. (2006) for our dataset as per your suggestion. The CFA results indicated that the model reasonably fits the data without a perfect match given the unique context of our study. We added the following paragraph to the Methods, statistical analysis section:

“*Moreover, a confirmatory factor Analysis (CFA) was conducted using STATA 14 software to verify the factor structure for MAPP-QOL hypothesized by Hill et al. [5]. The maximum-likelihood estimation method was employed, and a range of widely recognized goodness of fit indices were utilized to assess the adequacy of the model. The model fitness was assessed based on the Root Mean Square of Error of Approximation (RMSEA), Comparative Fit Index (CFI), Standardized Root Mean Square Residual (SRMR), and Chi-Square Statistic*”.

We also added the following paragraph to the Results section:

“*In the CFA, after modification of indices, the goodness of fit indices corresponding to the 5-factor model suggested in the original questionnaire indicated a reasonable fit with RMSEA= 0.052, CFI=0.847 and SRMR=0.062. We failed to reproduce the covariance matrix with a p-value <0.0001. Additionally, all items showed significant factor loadings on their respective factors. The lowest factor loading was observed for item 8 “Your surgical incision or episiotomy”, whereas the highest was observed for item 30 “Your ability to meet financial obligations”.*

In the Disucssion, we added the following statement:

“*In our attempt to examine the fit of the original factor structure suggested by Hill et al. [5] using CFA, the model seemed to approximate the data reasonably, yet it did not demonstrate perfect alignment with our observed data given the unique context of our study*”.

4. **Results: Baseline characteristics, Page 9, Line 191-192: The language description is too concise. Do you want to display a flowchart? The flowchart should be presented in the methodology section, and the flowchart is too concise, like a draft.**

Answer: Kindly note that Figure 1 displays a flowchart of the participants’ progress through the study. We also revised this sentnence as follows:

“*We recruited a total of 485 participants, of whom 24 withdrew by the second week, 11 withdrew between two weeks and one month, and 2 withdrew between one and three months, with an overall loss to follow up of 7.6% (Fig 1)”*

5. **Discussion: Paragraph 5, page 6: Please state the advantage of this study and the contribution to future research.**

Answer: We added the following sentences to the Conclusion:

“*This study provides the first Arabic instrument that can be used by clinicians to measure the QOL of Arab postpartum women, a period with relatively increased prevalence of significant social stressors and physical ailments that can negatively affect their QOL. Moreover, this valid instrument may bolster research efforts focusing on QOL issues in the Arab world. Future research is needed to replicate our findings in other Arab contexts, as well as to further refine the instrument for optimal fit with our cultural and contextual variations*”

We hope we have addressed all the comments of the kind reviewers in a clear manner, and revised the manuscript accordingly.

Thank you again for the thorough review of our paper.

Best wishes.

Sincerely,

Mona Nabulsi, MD, MSc

Professor of Clinical Pediatrics

Department of Pediatrics and Adolescent Medicine

Faculty of Medicine

American University of Beirut

Beirut-Lebanon

P.O.Box: 113-6044/C8

E-mail: [mn04@aub.edu.lb](mailto:mn04@aub.edu.lb)
